# Nonsurgical Procedures for Keratoconus Management

**DOI:** 10.1155/2017/9707650

**Published:** 2017-12-21

**Authors:** L. Rico-Del-Viejo, M. Garcia-Montero, J. L. Hernández-Verdejo, S. García-Lázaro, F. J. Gómez-Sanz, A. Lorente-Velázquez

**Affiliations:** ^1^Department of Optics II, Faculty of Optics and Optometry, University Complutense of Madrid, Madrid, Spain; ^2^Department of Optics and Optometry and Vision, Faculty of Physics, University of Valencia, Valencia, Spain

## Abstract

**Objectives:**

To describe the past 20 years' correction modalities for keratoconus and their visual outcomes and possible complications.

**Methods:**

A review of the published literature related to the visual outcomes and possible complications in the context of keratoconus management using nonsurgical procedures for the last 20 years (glasses and contact lenses) was performed. Original articles that reported the outcome of any correction modalities of keratoconus management were reviewed.

**Results:**

The most nonsurgical procedure used on keratoconus management is the contact lens fitting. Soft contact lenses and soft toric contact lenses, rigid gas-permeable contact lenses, piggyback contact lens system, hybrid contact lenses, and scleral and corneoscleral contact lenses form the contemporary range of available lens types for keratoconus management with contact lenses. All of them try to restore the vision, improve the quality of life, and delay surgical procedures in patients with this disease. Complications are derived from the intolerance of using contact lens, and the use of each depends on keratoconus severity.

**Conclusions:**

In the context of nonsurgical procedures, the use of contact lenses for the management of keratoconic patients represents a good alternative to restore vision and improve the quality of live in this population.

## 1. Introduction

Keratoconus (KC) is a bilateral but typically asymmetric, noninflammatory corneal ectasia characterized by progressive corneal thinning, conical protrusion, scarring, and decreased biomechanical strength of the cornea. The result is distorted and subnormal vision [1, 2]. Most recent epidemiologic studies determine that the age-specific annual incidence of KC is approximately 1 : 7500 or 13.3 new cases per 100,000, and the estimated prevalence of this disorder in general population is 1 : 375 or 265 cases per 100,000 [3].

KC pathogenesis has a multifactorial basis. The major environmental and behavioral risk factors seem to be a constant eye rubbing trauma causing a reduction in shear strength and cone-forming deformation [4] and the ultraviolet light as a precursor of the increased levels of oxidative stress markers and the decreased antioxidant capacity defenses in keratoconic corneas [5, 6]. Nowadays, there is sufficient data to suggest that KC has a strong genetic component [7].

KC negatively impacts on vision-related quality of life of affected patients making this disease a significant public health concern due to the economic burden associated with its management. The Collaborative Longitudinal Evaluation of Keratoconus (CLEK) study demonstrated that patients with this disease had lower scores on all National Eye Institute Visual Function Questionnaire scales except for ocular pain [8, 9].

KC management is often complex and varies depending on the state of progression of the disease. The approach includes the restoration of the normal visual acuity and/or correction of tectonic equilibrium of the corneal tissue. Rehabilitation of vision may be addressed from glasses in very early stages of the disease when regular astigmatism is present; the use of contact lens (CL) in more advanced cases or/and the application of different medical/surgical procedures when strengthening the cornea is also the target [10]. When extreme thinning or scarring is present, the condition warrants a corneal transplantation.

When irregular astigmatism is present, vision decreases dramatically. On the other hand, the evolution of the disease tends to be asymmetric, and even in the presence of regular astigmatism, the correction with glasses may result in aniseikonia due to the induced refractive anisometropia. In this situation, CL fitting, in its different forms, seems to be the best option. The process is often long and difficult for both the patient and the eye care practitioners. Comfort, good vision, and an adequate lens fit that does not compromise the cornea are the three most relevant targets [11].

Although this review is focused on the nonsurgical alternative to restore vision in the context of keratoconus management, in this sense, it is also important to consider a general recommendation for the patients not to rub their eyes, based on the relationship between the onset of the disease and the biomechanical impact of rubbing. For this reason, the use of topical antiallergic medication in patients with allergy or the use of preservative-free topical lubricants is recommended, especially in cases of ocular irritation [12].

Soft contact lenses (SCLs) and soft toric contact lenses, rigid gas-permeable contact lenses (RGPCLs), piggyback contact lens system (PBCLs), hybrid contact lenses (HCLs), and scleral contact lenses (SsCLs) constitute the contemporary range of available lens types for the KC management with CLs.

Curiously, most of the studies published on KC management with CLs are case reports and prospective case series, retrospective and prospective comparative type with the absence of any randomized controlled trial [10]. A review of the published literature related to the visual outcomes and possible complications in the context of KC management using any of the previously described forms of CLs is the purpose of this article.

## 2. Method

A literature search for publications in English and published in peer-reviewed journals was carried out using PubMed and encompassing the period from January 1, 1997, to June 1, 2017. The search equation was (keratoconus [mh] OR KC OR pellucid marginal degeneration OR PMD OR irregular astigmatism) AND (scleral contact lens OR scleral contact lenses OR corneoscleral contact lenses OR RGP OR rigid gas permeable OR rigid contact lens OR soft contact lenses OR silicone hydrogel OR toric soft contact lenses OR piggyback contact lens OR piggyback system).

The literature search retrieved 283 articles. The reference lists of these articles were also searched for any additional studies that were not identified by the electronic search. All the retrieved publications were reviewed, and some of them were excluded because they did not contain information of interest. Finally, 146 articles were selected and reviewed.

## 3. Results and Discussion

A variety of options, including both surgical and nonsurgical, are available for the KC management. While the surgical procedures alter the natural course of the disease, nonsurgical procedures pursue vision improvement. Our review is focused on nonsurgical procedures showing the available literature in the last 20 years.  Table 1 shows a classification of articles reviewed based on the type of correction used and type of study. The main correction used to improve vision in KC is the CL. As the severity of KC increases, CL fitting becomes more complicated, and special designs to match the altered corneal shape are required. In addition to KC severity, the type of CLs fitted depends on visual demand and CL tolerance of the subject.

### 3.1. Soft Contact Lenses (SCLs)

The role of SCLs for KC vision correction has changed in the past years. SCLs have comfort advantages over RGPCLs but tend to drape over the irregular KC cornea assuming the same irregular surface and resulting in poor visual acuity (VA). Early in the disease, SCLs with toric design may be adequate to correct myopia and regular astigmatism. SCLs and toric SCLs may be indicated in early KC, decentered KC, and RGPCL intolerance [13]. However, RGPCLs provided superior visual performances than SCLs.

In a study that enrolled 27 KC subjects, (severe KC (7), moderate KC (14), and mild KC(1)) the results showed that in the contact lens-wearing patients, RGPCLs gave significantly better low-contrast acuity (LCVA) compared to toric SCLs. Although high-contrast acuity (HCVA) scores were also found to be better with the patient's habitual RGPCLs (versus the toric SCLs), the differences were not significant. Nevertheless, with the exception of spherical aberration, toric SCLs were successful in significantly reducing uncorrected higher-order aberrations (versus spectacles) [14].

Nevertheless, SCLs designed specifically for KC have a useful role in early KC or where a patient might be intolerant to RGPCLs [15]. SCL designs for KC are thicker than conventional SCLs (from 0.3 mm to 0.6 mm). The greater central thickness of the CL helps prevent the lens from adapting to the irregular shape of the cornea. In this way, it has been proven that SCLs have a similar behavior to RPGCLs and thus can mask mild or moderate irregular astigmatism. However, a greater thickness causes a decrease in the oxygen permeability (DK) of the lens, a situation that encourages the development of possible complications. Lens designs have adjustable peripheral curves that allow adjusting the movement and adjustment of the lens regardless of the base curve. The peripheral zone becomes thinner, thus improving the DK and overall comfort. These lenses are available in high spherical and toric powers commonly required for KCs. Some SCL designs for KC are available in reverse geometry, and silicone hydrogel (SiH) lenses are available in some keratoconic designs.

Some of the special designed SCLs for keratoconus are HydroCone® (Toris K) (SwissLens, Prilly, Switzerland) and KeraSoft® IC (Bausch & Lomb Inc., Rochester, NY).

Soft HydroCone (Toris K) lens is made of SiH material Silikon-Hydrogel (definitive 74%, Igel 77%) that includes a front toric surface and has a dynamic stabilization with nasal and temporal bumps. Basically, there are 2 types of lenses in a trial set: HydroCone-K12 (for grade 1*-*2 KC) and HydroCone-K34 (for grade 3*-*4 KC). These lenses can be categorized as custom soft contact lenses (CSCLs) because both spherical and cylindrical corrections are added to the toric surface of the lens to increase visual performance [16].

A comparative study with Toris K lens versus spectacle correction or without correction in 55 KC eyes showed improvement in VA and some aberrations (coma and trefoil). The mean increase in VA was 4.5 lines (range 1–9 lines). Furthermore, point spread function (PSF) was significantly better with the lens, meaning much better image quality [16].

On the other hand, there is no improvement in VA when toric K lenses are compared with RGPCLs [13, 17], and even the quality of life, measured with the CL impact quality of life (CLIQ), is similar [18]. It is expected that SCLs improve visual function in KC eyes [16, 19], but the challenge is to achieve visual results similar to those provided by RGPCLs.

KeraSoft IC is a custom-lathed, front-surface toric soft silicone hydrogel (Efrofilcon-A, water 74%). The periphery of lens can be manipulated independently of the base curve with two different geometries: full periphery and sector management control, which is a quadrantic-specific design that can be individually customized for the periphery, making it able to conform to the shape of any cornea. This model is a good alternative for the optical management of irregular corneal astigmatism in nonsurgical corneal ectasias such as KC and pellucid marginal degeneration (PMD). The results of a retrospective study of 94 eyes fitted with the SiH KeraSoft IC lens were compared with 77 eyes fitted with Rose-K2 RGPCLs (Menicon); VA outcomes were reported to be similar with both lens modalities for patients with mild and moderate corneal ectasia [20].

Such designs are used in the management of irregular cornea (e.g., postkeratoplasty [13] and traumatic keratopathy [21]). Toris-K lens is fitted in this case report results. Further prospective, randomized, and controlled studies are needed to confirm these results.

In cases of KC treated with KeraRing intracorneal ring segments (ICRS), these patients can obtain better levels of visual acuity than with spectacles and an acceptable wearing time with a low incidence of complications. Clinically, post-ICRS cases might need more sector management control lenses and steeper peripheral designs due to the profound alterations the devices may induce in the cornea. The results were obtained in 32 eyes fitted with KeraSoft IC [22]. Carballo-Alvarez et al. [23] published their results in patients with the same type of ICRS, but who were fitted with lathed toric SCLs. Their results showed that SCLs are a viable option for good vision and comfort in a significant proportion of KeraRing ICRS-implanted eyes. In eyes in which this type of CLs proves to be inadequate, a piggyback system (PBCLs) could be a satisfactory option.

High order aberrations (HOAs), especially coma-like aberrations, are significantly higher in eyes with KC than in normal eyes [24]. Most of the KC eyes have vertical coma where the wavefront is fast in the superior portion and slow in the inferior portion due to a superior-inferior asymmetry of the shape of the cornea in eyes with KC [18, 25].

Some authors explored the correction of HOAs with CSCLs, such as posterior [26, 27] or anterior surface-customized lenses [28, 29].

Suzaki et al. [26] devised a standardized asymmetric SCLs for patients with KC that had asymmetric diopters in the posterior surface and performed a preliminary study to investigate the feasibility of their prototype lens that had no sphere or cylinder power. So, spectacles were used in addition to the CLs to evaluate visual performance. The prototype lens was made of a SiH (asmofilcon A). The design was developed to correct the vertical asymmetric pattern seen on corneal tomography. The peripheral design of the lens was a double vertical asymmetric slab-off and horizontal right and left ballast. The lens diameter was 14.5 mm and its central thickness was 0.15 mm [26]. They included 30 eyes (26 patients) with KC and compared the visual performance and optical quality with their SCLs and without SCLs. They concluded that the use of standardized lenses with spectacles resulted in statistically significant improvements in corrected VA and all the endpoints for HOAs when compared with spectacles without their lenses. However, the decentration of the resting position of the lens was associated with an increase in horizontal coma and reduction of correction. In spite of the results obtained by the authors, the improvements were assessed in relation to an eye without any type of compensation of KC and eye with fitted SCLs.

Other authors compare between subject's habitual RGPCLs and the CSCLs designed by them, although they only present results for three subjects with KC grades 1, 2, and 3. Marsack et al. [27] fitted the toric posterior surface-customized lenses defined by the subject's corneal astigmatism determined by topography. The spherical anterior surface was based on the spherical power that was determined from the subject's habitual correction. The stabilization system was based in prism ballasting to minimize rotation, and the material used was silicone hydrogel (methafilcon A). Their results broaden clinical observations of the CSCLs versus the subject's habitual RGPCLs. Although only focusing on three keratoconic subjects with RGPCLs and comparing the performance of both corrections, HCVA LogMAR with CSCLs was equivalent to the usual RGPCL corrections in the laboratory terms.

Table 2 summarizes the characteristics and results of some of the most relevant studies on KC management SCLs.

Other authors have worked on the design of anterior surface-customized lenses. Chen et al. [28, 29] designed a model of CSCLs whose back-surface profiles are sculpted to match the anterior corneal surface of KC eyes. They compared lens stability, aberrations, and visual performance in three KC eyes between conventional SCLs and CSCLs. Anterior corneal topography data for a central 5 mm diameter was used to design an ablation profile for the same diameter around the CL center. Prism-ballasted conventional toric SCLs manufactured with 45% water content hydrogel material had 14 mm diameter and 182 *μ*m center thickness. Although the back-surface CSCL compensated for most of the anterior corneal aberrations, significant residual HOAs contributed by the posterior cornea and the crystalline lens still remained. In particular, the posterior cornea and crystalline lens contributed 56% and 41%, respectively, to the measured overcorrection of vertical coma in eyes with the back-surface CSCL on average [28]. Lens stability was better for CSCL. Lens movements with conventional SCLs were significantly stabilized with the back-surface customization. This behavior resulted in an improvement of in HCVA and LCVA, even in the presence of imperfections in lens fabrication and typical amounts of lens decentration [29]. The authors indicate that CSCLs have the potential to provide KC eyes a normal level of vision.

It is well known that some movement of the lens is required to allow tear exchange under the lens and thus to provide lubrication and essential nutrients to the cornea. Therefore, the search for the balance between the necessary movement and decentration of the lens is the goal of some designs. Using optical simulation, de Brabander et al. [30] showed that visual performance declined when the decentration of a custom-made SCL exceeded 0.5 mm. The impact of translation and rotation of “ideal” customized corrections in three typical clinical cases of mild, moderate, and severe KC was explored, theoretically, by Jinabhai et al. [18]. Optical quality of the eye was assessed in terms of its wavefront aberration and PSF. They compared with the RGPCLs corrections and the results only provided optical improvements if the movements were constrained.

The challenge is to achieve comfortable materials and designs that ensure corneal integrity and in turn provide a visual quality similar to that of RGPCLs.

### 3.2. Rigid Gas Permeable Contact Lenses (RGPCLs)

RGPCLs have been long used for the correction of ametropia or corneal irregularities [31]. After the development of hydrogel materials, and especially after the development of SiH materials, corneal rigid contact lenses gradually became a second line solution when SCLs did not provide good enough quality of vision or in cases of irregular cornea [26].

Overall, most CL patients are fitted with SCLs and the use of RGPCLs remains limited. Frequent indications for RGPCL are KC, PMD, postsurgical ectasia, irregular astigmatism, or orthokeratology (OK).

According to their total diameter, RGPCLs can be classified as corneal (8–10 mm) and intralimbal (10.5–12 mm) lenses [32]. The largest diameters (12 mm) of the intralimbal group are also known as pancorneal CL [33]. Total diameters over 12 mm can be divided into semiscleral and scleral designs [32], as these CLs bear partially or totally on the sclera.

Another basic parameter of a CL is its base curve. Attending to this detail, RGPCL fitted the range from standard monocurve, bicurve, or multicurve lenses [34, 35] to reverse geometry lenses [36] or specific curvature designs such as those developed by Rose [37] or implemented in the IKone lenses [31].

The uses of RGPCLs vary from orthokeratology to irregular corneal vision restoration and refractive error compensation. The latter was widely accepted since rigid CL allowed a better oxygen interchange due to faster tear exchange behind the lens than SCLs [38].

However, invention of SiH materials more than a decade ago [39] has changed this picture [38], and today, disposable SiH SCLs are the first choice for those patients requiring refractive correction alone.

With this idea in mind, it is immediate to think that RGPCLs are being limited to orthokeratology and vision rehabilitation in those cases where good quality of vision cannot be achieved with SCLs. In a study over 27 KC patients in 2013, Yildiz et al. concluded that the type of CL—soft or RGP—may not have an impact on the quality of life [17]. However, other more recent studies demonstrate the opposite [40]. Moreover, Bilgin et al. demonstrated in a sample of 518 KC patients in Turkey that the use of RGPCLs delayed the need for surgery in 98.9% of the cases [41].

The mechanism of action of RGPCLs and rigid (usually PMMA) CLs on KC has been known for many decades [31] and is quite simple from an optical point of view: lenses act as a mold for the tear film layer to cover corneal irregularities [42].

Since CL materials, the tear film layer and cornea have a similar refractive index, and HOAs are almost totally compensated, giving the patient a significantly higher quality of vision, although a residual uncorrected wavefront error remains [43]. It is estimated that the amount of HOA correction is around ninety percent [42].

Asymmetrical corneal protrusion causes a myopic shift and irregular astigmatism [31], which is composed—among others—of coma-like aberrations. In the human eye, coma is characterized by an asymmetrical distribution of optical power within the pupil. Typically, KC and PMD show a curvature increase in the lower half of the cornea coupled with a flattening of the upper half, which translates into an irregular wavefront that causes image distortion [44].

Any irregular cornea can potentially benefit from the use of RGPCLs [32, 45–47], but since corneal RGPCL diameter is smaller than white to white and they tend to center over the apex [33], central and paracentral irregularities are the best cases for this type of lenses.

For those eyes with decentered apex, ocular surface disease or corneal surgery, either intralimbal or scleral designs, is preferred [48–50].

Quality of vision improves for the patient. Oie et al. reported the use of RGPCLs to achieve better intraocular structure visualization during cataract surgery in two cases of severe KC [51], and Uzunel et al. [52] recommended irregular astigmatic correction with RGPCLs in eyes with KC before performing an optical coherence tomography (OCT).  Table 3 summarizes the characteristics and results of some of the most relevant studies on KC management RGPCLs.

Despite being described for a long time, irregular cornea management remains a challenge for eye care practitioners [53] and new technologies are applied to develop tools and algorithms that assist decision making to reduce the total number of trial lenses and visits to the clinic [54–59] or choose the best management—from glasses to surgery—for a particular patient [60].

While RGPCLs have been long used for KC vision restoration and are undoubtedly useful, they have inherent risks and induce corneal and tear film layer changes.

Notwithstanding initial discomfort, many patients wear RGPCLs for several hours each day [45]. Ocular surface and tear film alteration associated with the use of CLs have been previously described in scientific literature [61–63]. Changes in KC patients might be associated with CL use, rather than with the pathology itself [64]. Lema et al., in a study over 88 subjects, described an overexpression of proinflammatory cytokines in the tear film layer of KC patients wearing RGPCLs [65]. Fodor et al. extend this conclusion to all types of CLs, confirming a higher concentration of proinflammatory agents in tears also in SCL users [66]. Carracedo et al. proved that not only KC patients present greater signs and symptoms of dry eye and tear instability [67] but RGPCL use in KC also increases the concentration of Ap4A in tears, which is related to dry eye [68].

Ghosh et al. used confocal microscopy to demonstrate that corneal RGPCLs reduce anterior and posterior stromal keratocyte density, increase anterior stromal keratocyte cell area, and induce endothelial pleomorphism, but no polymegathism or endothelial cell density was detected after one year of use [69]. Bitirgen et al. proved in 2013 with the same technology that KC causes corneal microstructural abnormalities and that RGPCL wear is associated with reduced basal epithelial cell and anterior stromal keratocyte density. No effect was found on endothelial cell density. Contrary to Ghosh et al., other authors found no effect on posterior stromal keratocyte density [70]. Another study by Erie et al. also using confocal microscopy confirmed apoptotic death of keratocytes in RGPCL wearers compared with toric SCL users, probably due to cytokine release after epithelial injury [71]. Edmonds et al. reported lower endothelial cell count in keratoconic corneas using SoftPerm® hybrid contact lenses, but found no differences among the non-CL users or those eyes wearing SCLs or RGPCLs [72].

From a morphological point of view, Hwang et al. confirm that the use of properly fitted multicurve RGPCLs has no effect on KC progression [35].

Corneal curvature modification due to RGPCL use is a benign situation that must be detected and reverted [73–75]. Potentially, any CL can induce corneal warpage [76], but the risk is higher with corneal RGPCLs due to their material properties, design, and fitting technique [77].

Time seems to be a crucial factor for corneal warpage and refractive changes associated with it, since early RGPCL users do not present topographic signs [78], and refractive state [79] and root mean square (RMS) changes in time after suspending CL use [80].

While RGPCLs have been long used for KC vision restoration and are undoubtedly useful, they have inherent risks and induce corneal changes.

Moderate and severe cases of corneal irregularity frequently complain about foreign body sensation and limited wear time due to discomfort [81] that reduces quality of life. The use of Acular® to reduce painful sensation due to CL does not seem to be effective [82].

CL intolerance due to corneal nodule formation in KC might be successfully treated performing anterior stromal puncture of the cornea [83] or phototherapeutic keratectomy [84].

Immediate use of RGPCLs after corneal crosslinking (CXL) might delay sub-basal nerve plexus regeneration and cause epithelial cell stress [85]. Ünlü et al. reported better tolerance to RGPCLs for at least six months after CXL, probably caused by a decreased corneal sensitivity and flattening of the cornea [86].

Patients undergoing corneal transplantation should be carefully examined after surgery since we could be facing a completely new eye. Graft characteristics might contraindicate corneal RGPCL readaptation [87], and in some cases, scleral RGPCLs or PBCLs might be the first choice.

### 3.3. Piggyback Contact Lens (PBCL) System

Introduced in the early 1970s [88–91], the piggyback contact lens (PBCL) is a system composed of two CLs, a RGPCL and a SCL that are placed together on the eye. This modality is normally indicated in patients that experience RGPCL intolerance or discomfort, significant corneal staining due to an unstable RGPCLs, presence of scar, or even epithelia basement membrane dystrophy [32, 92]. SCL is placed first, covering the whole cornea and providing a bandage effect that helps in the protection of the KC apex and in a better RGPCL stabilization [91]. For this reason, patients experience better comfort, increased wearing time, and similar VA in comparison to RGPCL alone [93]. Regarding PBCL fitting, most of the practitioners [92–94] usually use a low-positive-powered SCL since it is believed to facilitate RGPCL centration, and also, it does not contribute to the total power of the PBCL system [95].

On the contrary, Romero-Jimenez et al. [96] reported that the use of a mild negative-powered SCL allows the fitting of a flatter and less powered RGPCL leading to better centration and movement due to the lighter weight of the RGPCL. Besides, it has a relevant impact on the oxygen transmissibility that makes it more appropriate for piggyback system. Normally, in order to obtain an optimal fitting, both CLs need to move independently and correctly at each blink observed by slit lamp and an acceptable fluorescein pattern with no touch [97]. It has been reported that only 2% of the KC CL wearers use PBCL [98].

The low-DK materials available and fitted in the beginning might be behind of this low rate. Two of the most reported PBCL complications are the difficulty to handle two CLs and the low DK, which acts as a double barrier over the corneal surface [99]. High-DK CL materials were developed in order to reduce, in part, the corneal hypoxia and corneal edema-related complications derived from this modality. Therefore, CL with high DK is needed to ensure corneal health and patient comfort [94]. The most used combination for PBCLs system reported in the literature is a combination of SiH SCL and a high-DK RGPCL. An in vitro study conducted by Lopez-Alemany et al. [100] demonstrated that the optimum PBCL system is obtained with a combination of a SiH SCL with a high or moderate DK RGPCL. In this sense, O'Donnell and Maldonado-Codina [94] refitted successfully a 54-year-old woman that was intolerant to RGPCL with a hypertransmissible hydrogel-RPG piggyback system (Focus Night & Day (CIBA Vision) and Menicon Z material (Menicon Co., Nagoya, Japan), resp.,). Six months after the fitting, the patient was highly satisfied with the comfort and vision provided by the CLs. In addition, corneal vascularization and limbal and bulbar redness were markedly reduced in comparison to her previous CL (SoftPerm lenses). Other study conducted by Sengor et al. [92] fitted a PBCL (lotrafilcon A and fluorosilicone methacrylate RPG copolymer, resp.) in 16 patients that were CL intolerant. Participants showed better VA outcomes in comparison with those with spectacles, and no signs of hypoxia such as hyperemia, vascularization, or corneal edema were observed during the study.

As well, successful results were obtained by Mehta et al. [101] in patients with irregular corneas after PBCL fitting (8 of the total sample were KC patients) regarding optical and clinical performance.

Despite the results before mentioned, Acar et al. [97] examined the immune histochemical effect of the PBCLs during 6 months of wear in patients with KC that never wore CLs before the study. The PBCL system used was SiH-balafilcon A (PureVision, Bausch & Lomb, Rochester, NY) and fluorosilicone methacrylate copolymer (CFA, 100 UV, Zeiss, Germany), respectively. After performing conjunctival cytology, interleukin tear film (IL) measurement, and confocal microscopy, the authors found an increased IL levels and a significant decrease in posterior keratocyte density after 6 months of wearing. Despite the small sample, these findings could have important implications since these patients need to wear CL for long term and they are more predisposed to ocular surface damage.

Furthermore, PBCLs have been used over intrastromal corneal ring segments (ICRS, Intacs®) in patients with KC. Hladun and Harris [102] first described a successful case fitting the 1-day Acuvue (Johnson & Johnson K.K., Japan) disposable SCL with a Burger Kone® keratoconic RGP design. The patient obtained an acceptable comfort and VA.

Similar results were obtained by Smith and Carrell [103] that fitted a PBCL (balafilcon A and paflufocon B, resp.) in a 41-year-old man with a progressive and advanced KC in both eyes. On the contrary, Ucakhan et al. [104] tried to fit PBCLs with balafilcon A (PureVision, Bausch & Lomb, Rochester, NY) and an aspheric RGPCL but despite the good vision achieved, the patient refused to use it. Recently, Kumar et al. [105] quantified short-term changes in corneal HOAs with PBCLs in an eye with Intacs. They found that after PBCL fitting, VA improved and visual symptoms were reduced (it had been observed objectively with a decrease in cylindrical power with reduced HOAs along with improvement in PSF). In all cases, the main objective was to provide a good vision to the patient without compromising their ocular health before/after KC surgery.

Table 4 summarizes the characteristics and results of some of the most relevant studies on KC management PBCLs.

### 3.4. Hybrid Contact Lens (HCL)

Hybrid contact lenses (HCLs) combine a rigid central zone and a soft peripheral skirt. This type of CL tries to exploit the best features of RGP (better quality of vision) and soft (the comfort and ease) materials. HCLs emerged as an alternative option to PBCLs and RGPCLs [106, 107].

Currently, the HCLs available in the market represent the evolution of the first HCLs such as Saturn II (OPSM, Contact Lenses, USA) and the SoftPerm lens (Sola/Barnes-Hind Incorporated) [108], that were an attempt to provide a better alternative for KC patients. Many of the complications derived from the predecessor such as the problem with the comfort, the low-DK [109], and the durability of the rigid-soft interface have been improved in the new generation. These are made of high-DK materials, and also, there are many designs for specific applications that allow the practitioner to achieve outstanding outcomes in cases of regular astigmatism [110] and irregular corneas [107].

The SynergEyes® (SynergEyes Inc., Carlsbad, CA) HCLs were developed with a base-curve design (KC), stronger RGP/hydrogel junction, and higher DK of the central zone. Optimal fitting is achieved when complete apical clearance exists (without air bubbles) with a soft landing of the lens at the junction zone [111]. In this design, a steeper skirt increases the lens movement, avoiding lens adherence.

Concerning ClearKone®, the other HCLs available (which will be replaced by the UltraHealth that integrates a silicone-hydrogel skirt) show a reverse-geometry design that requires fitting vault and skirt curvature separately [111]. The use of high-molecular weight sodium fluorescein is crucial for the fitting process in this type of lens.

Nowadays, research studies related to HCLs and their clinical performance are still limited. Among the studies carried out, the performance of the SoftPerm lens was evaluated in a retrospective case series of 14 patients (24 eyes) with KC [108]. The best-correction visual acuity (BCVA) with SoftPerm lens significantly improved (83.3%) compared to that with spectacles. However, many complications were reported during HCL wear such as giant papillary conjunctivitis (25%), nonspecific ocular discomfort (29.1%), lens damage (29.1%), and corneal vascularization (25%). Regarding the new generation of HCLs, Abdalla et al. [112] reported that 87 percent of the patients of the study (with KC and PMD; 61 eyes) were successfully fitted with SynergEyes KC HCLs. In addition, Nau [107] found that 79.5% of the patients with irregular cornea (79 eyes) experience higher comfort with SynergEyes KC HCLs than RGPCLs. Clinical and quality of life outcomes have been evaluated in patients with KC after fitted with ClearKone HCLs and PGBCLs [106]. Despite the fact that the VA did not differ between these two modalities, the ClearKone HCLs showed higher vision-related quality life scores. Similarly, Carracedo et al. [113] compare the clinical performance of this HCL against the habitual CL of the patients. They found that the ClearKone provides an improvement in VA, contrast sensitivity, and subjective comfort when compared with the other CLs for patients with KC.

Fernandez-Velazquez [114] reported three case series where the early wear of ClearKone HCLs provokes circular corneal clouding. Notwithstanding high VA and comfort reported by the patients, the appearance of this phenomenon leads to a discontinuation of the HCLs in order to avoid severe complications. Furthermore, immunohistochemical changes have been studied in patients with KC fitted with ClearKone HCLs and PBCLs during 6 months [97]. The authors reported that no significant changes in IL levels and confocal microscopy were found between these two modalities. In light of these results, more research studies focused on the impact of these CLs on the ocular surface in long-term are needed in order to know the implications of fitting this modality.

Table 5 summarizes the characteristics and results of some of the most relevant studies on KC management HCLs.

### 3.5. Corneoscleral and Scleral Contact Lenses (C-ScCLs and ScCLs)

Corneosclera (C-ScCLs) and scleral contact lenses (SsCLs) (mini or large scleral) are made using high oxygen-permeable materials [115]. These advanced materials together with the fact that the bearing zone is on the sclera (full or partially) [116] present these CLs as the ideal designs for KC and allow the eye care practitioner in selecting the optimal fitting parameters. The main parameter in this fitting modality is lens diameter. The larger the diameter, the greater the distance between posterior contact lens surface and central cornea (vault) is. Therefore, the postlens tear meniscus created enables fitting these lenses in the eyes with the most severe degrees of KC. The fitting success depends on the right combination of this parameter, contact lens DK/t, and corneal thickness.

C-ScCLs and ScCLs are not usually the first choice to fit but are commonly prescribed when other CLs (SCLs, RGPCLs, PBCLs [94], and HCLs [106, 107, 112, 113]) show tolerance problems [117, 118] or do not provide acceptable VA. [48–50, 118–121].

C-ScCLs and mainly ScCLs (mini and large scleral lenses) have been fitted in ectasias, both primary ectasias (KC [121–131] and PMD [124, 127, 132]) and iatrogenic ectasias (postrefractive surgery ectasia [124, 126]), postpenetrating keratoplasty [121, 123, 128, 129], irregular cornea [132], irregular astigmatism [123, 127], ocular surface disorders [122, 123, 125, 127, 133], and severe dry eye syndrome [126, 134].

In general, the indications for these types of contact lenses are vision improvement, ocular surface protection, and cosmetics/sports indications. Specifically, two are the main goals for C-ScCL and ScCL fitting in KC patients. The first and most important is visual improvement and the second is CL tolerance improvement.

The sclera is the ocular structure where ScCLs rest (mini and large scleral) while C-ScCLs rest partly on the cornea and partly on the sclera. Thus, the optimal meniscus thickness changes depending on the CL diameter. The postscleral lens tear meniscus allows the SsCLs not to touch the limbus and cornea, and therefore, the fitting of these lenses is independent of the corneal shape [116], offering different advantages: good centration, stability, and improved VA.

These advantages differ depending on the type of CL design. C-SsCLs, due to a larger optical zone, provide more consistent visual performance and greater on-eye stability than corneal contact lenses [135]. However, optical and visual performance offered by SsCLs is better than that provided by C-SsCLs. SsCLs contribute to a decrease in HOAs in KC patients. HOA reduction is significant in comparison with optical correction by spectacles or hydrogel CLs and lower when compared with corneal RGPCLs [135]. Downie [136] and Marsack et al. [137] documented the capability to correct HOAs with wavefront-guided scleral contact lens. This customized fitting allows presenting normal HOA levels and significant improvement vision in advanced keratoconus patients. Rotational and translational stability of scleral lenses may provide an ideal platform for the correction of highly aberrated eyes.

SsCLs offer a very stable platform for adjustment of design to improve visual quality. Patients with KC have shown an 88% improvement in visual acuity postscleral lens fitting [120]. In an observational retrospective study, Picot et al. [118] evaluated the contribution of scleral lenses in terms of improving the quality of life in the treatment of astigmatism after penetrating keratoplasty and keratoconus. In this study, VA progressed from 0.68 ± 0.46 to 0.15 ± 0.17 logMAR at the 6th month after ScCL fitting in the better eye. This improvement in terms of VA has been reported in different studies. Baran et al. [124] found that 93% of patients had VA higher than 20/40 when baseline was 20/70. In the same way, Rathi et al. [125] observed that 75% of patients improved more than 2 lines of visual acuity. Recently, Yan et al. [117] reported an improvement from 0.88 logMAR to 0.10 logMAR in KC patients using mini SsCLs.

Nevertheless, several studies show a decrease in visual performance when SsCLs was fitted. Mini or large SsCLs need to be filled with ophthalmic solution to prevent air bubble formation in the postlens tear meniscus. If the appropriate one is not used, this ophthalmic solution will increase postlens tear layer turbidity. In this sense, Carracedo et al. [131] reported that VA decreases when wearing scleral lens filled with preserved saline solution. In a separate study, VA decreased by 45% after 4 hours due to tear debris [125].

The major advantage of these designs compared to RGPCLs is improved comfort. With greater diameter, CL stability will be higher and so will the vault. In general, there is an agreement that mini and large SsCLs, when settled onto the eye, should display a central clearance of around 200 *μ*m and 75 *μ*m at the limbus [138]. Edrington et al. [139] found that minimal peripheral clearance is associated with reduced lens comfort. For this reason, the absence of this movement and considerable central and peripheral clearance, mainly in mini and large SsCLs, provides a good comfort and tolerance to these lenses. One clear example of this was the Yan et al. [117] study where all eyes included were reported as comfortable at initial use of mini SsCLs, and 91% were comfortable after a 3-month follow-up. Similar values were described previously by Pecego et al. [121] for a 3-month follow-up, reporting good tolerance in 77% of eyes fitted with Jupiter scleral lenses. This is reflected on the quality of life in patients wearing SsCLs where average scores on the NEI-VFQ 25 questionnaire of 80.2/100 with SsCLs versus 48.1/100 without SsCLs [118] have been reported.

Table 6 summarizes the characteristics and results of some of the most relevant studies on KC management ScCLs.

Corneal vaulting, centration, and excellent comfort have allowed expanding the use of C-SsCLs and mini SsCLs to less severe KC cases. These scleral-lenses attribute plus larger diameter can be an optimal scenario to incorporate more complex optical designs (customized visual corrections or multifocal designs for presbyopia).

Mini ScCLsl and, mainly, large SsCLs are the preferred designs for fitting severe irregular corneas or postkeratoconus surgery (CXL, ICRS, or penetrating keratoplasty). In these cases, where there are important corneal elevation and severe corneal irregularities and asymmetries or if the goal is protecting the ring/suture area from touch, high vault is also required. These fittings have shown stable vision and good tolerance [140–143].

SsCL fitting has been reported as an effective and safe alternative for managing KC patients [143]. However, there are studies that show low incidence of nonsevere adverse events, thus visual disturbance (halos and haze), discomfort or pain, or, simply, difficulties with the lens insertion or removal [121]. Only severe adverse event case reports have been described in KC patients [144], and it is unclear if these events are related to the pathological corneal condition.

CLs are the usual option chosen by the specialist to KC management as a nonsurgical treatment. SCLs, RGPCLs, PBCLs, HCLs, C-SsCLs, and SsCLs form the contemporary range of available lens types for the management of KC with CLs. All of them provide comfort and a restored vision after being fitted. Because KC is a progressive disease, the use of each one depends on the KC severity and CL fitting becomes more complicated. For this reason, special designs to match the altered corneal shape are required. At the beginning of the disease, toric SCLs and RGPCLs are the best option. In general, SCLs are the first option to fit before trying another type of CLs. In these cases, patient achieves good visual acuity with spectacles or soft toric CLs. RPGCLs are used as second option due to an important improvement in quality of vision when the SCLs cannot do it. When these CLs are not tolerable or the outcomes in vision are not enough to have a good quality of life, it is necessary to attend to another type of CLs such as PBCLs, HCLs, and ScCLs. PBCLs increase wearing time and improve vision and comfort but problems to handle two CLs and a high risk of hypoxia (Table 7) make them a nondefinitive system to use. HCL is an alternative to this one. The design resolves problems of manipulation and hypoxia but more research studies focused on the impact of these CLs on the ocular surface in long-term are needed. Finally, ScCL design provides a good level of comfort and vision when the disease has progressed.

Complications associated with CL fitting in KC management are shown in Table 7. RPGCLs present the most negative effect over the ocular surface (corneal warpage, negative effect over the tear film and others), even if the best results in optical quality of vision are achieved with these CLs. SsCLs are the ones that provide the best results in comfort and vision when the disease has progressed. In fact, only complications with the tear firm turbidity or problems with the manipulation have been reported in the reviewed literature (see Table 7). In cases where vision cannot be re-established with these CLs, surgical methods will be necessary. Another important point of view is the use of these CLs after KC management with surgical methods. Ablatives procedures, ICRS implantations, and CXL can be used to avoid the corneal transplantation when CLs are not enough to keep the integrity of the cornea and restore vision [145]. After these procedures, CLs can be prescribed to protect the ring/suture area from touch. In these cases, mini ScCLs and large SsCLs are preferred since they provide a more stable vision and better tolerance.

In summary, the use of contact lenses in KC management provides the best visual rehabilitation and improves patients' quality of life. Because several designs are available, different lenses could be fitted depending on the severity of the disease (early stages with soft or corneal GP lenses; mild-moderate with corneal GP lenses, and severe with scleral and corneoscleral contact lenses), helping to delay surgical procedures. Finally, in cases that require some surgical option (intracorneal rings or keratoplasty) but do not achieve good visual acuity, GP lenses (corneal, corneoscleral, or scleral designs) are a good alternative to improve postsurgical visual acuity.

## 4. Conclusions

In the context of nonsurgical procedures, the use of contact lenses for the management of keratoconic patients represents a good alternative to restore vision and improve quality of life in this population.

## Figures and Tables

**Table 1:** Classification of articles based on the type of intervention and type of studies.

Corrections	Numbers and type of study					
	Prospective comparative	Prospective cases series	Retrospective	Case report	Total	Follow-up range
SCLs	4	8	4		16	Two weeks–24 months
RGPCLs	10	7	4	1	22	3 months–25 years
PBCLs	4			5	9	3 weeks–3 years
HCLs	2	1	4		7	1 day–23 months
SsCLs	6	11	12	3	32	1 day–85 months

SCLs: soft contact lenses, RGPCLs: rigid gas permeable contact lenses, PBCLs: piggy back contact lens system, HCLs: hybrid contact lenses, SsCLs: scleral contact lenses.

**Table 2:** Description of the main studies in keratoconus (KC) management with soft contact lenses (SCLs).

Author	Type of study	Type CL	Sample size (eyes)	Severity KC (number of cases)	Follow-up (months) (mean)	Mean age (years)	Sex (female/male)	HOA (*μ*m)	UCVA (logMAR) (mean)	BCLCVA (logMAR) (mean)
Gumus and Kahraman [16]	Prospective comparative	SCL SiH (Toris K)	50/20^∗^/30†	Moderate (50)	2 Weeks	24.5	32/18	0.71^∗^0.49†	NR	0.00
Fernandez-Velazquez [20]	Retrospective	SCL SiH (KeraSoft IC)	94	Mild (22) Moderate (46) Severe (6)	10.3	35.5	NR	NR	NR	0.04
Suzaki et al. [26]	Prospective cases series	SCL SiH	30	Moderate (30)	NR	34.7	6/24	0.56	NR	−0.11
Sultan et al. [13]	Retrospective	SCL SiH (Toris K)	64	Mild-moderate (46) Severe (10)	11.94	27.92	26/24	NR	0.83	0.20
Yildiz et al. [17]	Prospective case series	SCL SiH	24	Mild (6) Moderate (13) Severe (5)	4	31.7	4/8	NR	NR	0.10
Yilmaz et al. [19]	Retrospective	SCL SiH	60	Moderate (60)	24	27.3	18/23	NR	0.85	0.16

UCVA: uncorrected visual acuity; BCLCVA: best contact lens corrected visual acuity; HOA: high order aberration; CLIQ: contact lens impact on quality of life questionnaire; CXL: corneal cross-linking; NR: not reported. Mild KC (average sim *K* < 45 D); moderate (average sim *K* 45–52 D); severe (average sim *K* > 52 D). ^∗^No previous CXL. ^†^Previous CXL.

**Table 3:** Description of the main studies in keratoconus (KC) managed with rigid gas permeable contact lenses (RGPCLs).

Author	Type of study	Sample size (eyes)	Severity KC	Follow-up (months) (mean)	Age (years) (mean ± SD)	Sex (male/female)	UCVA (logMAR)	BCLCVA (logMAR)	Comfort level	Other findings
Zadnik et al. [98]	Cross-sectional, observational	2418	Mild (110 eyes), moderate (1172 eyes), severe (1130 eyes)	NR	39.3 ± 10.9	55.9%/44.1%	Entrance VA. (Patients) 0.0 or better: 409 >0.0 to 0.3: 693 >0.3 to 0.54: 84 >0.54 to 1.0: 18 >1.0: 18	(Patients) 0.0 or better: 538 >0.0 to 0.3: 612 >0.3 to 0.54: 43 >0.54 to 1.0: 10 >1.0: 1	72.8% comfortable 27.1% irritating at least one eye	SCL: 4% RGPCL: 73% Piggyback: 2% Hybrid: 3% Eye rubbing: 50.4% Vogt's striae: 65% Fleischer's ring: 86% Corneal scarring: 53% Corneal irregularity: 99.7%
Bilgin et al. [41]	Retrospective, cohort	1004	Mild (5 (47%)) Moderate (4 (5%)) Severe (40.1%)	18–30	34.8 ± 10.1	52.7%/47.3%	0.62 mild 0.70 moderate 1.30 severe	0.10 mild 0.10 moderate 0.22 severe	New adaptation due to intolerance and irritation: 32.7% mild KC 23.7% moderate KC 17.6% severe KC	PKP: 2.0% Acute hydrops: 0.3% Unilateral KC: 10.6% Cone like shape: 20.6% Subepithelial opacity: 14% Descemet folds: 8.6% Fleischer's ring: 1.8% Munson's sign: 6.2% Vogt's striae: 7.5%
Hwang et al. [35]	Prospective, case–control	107	Mild (3.7%) Moderate (43.9%) Severe (52.3%)	22.6	25.4 ± 6.8 cases 22.3 ± 7.7 controls	53.3%/46.7%	Spectacle corrected 0.68 cases 0.54 controls	−0.032 cases	87% comfortable 9.1% slightly uncomfortable 3.9% uncomfortable	
Kamar et al. [33]	Prospective, cohort	30	Mild (4 eyes) Moderate (14 eyes) Severe (12 eyes)	22	48.5 ± 9.9	50%/50%	Contact lens corrected 0.20	Pancorneal RGPCL 0.15	6.6% intolerance	Improvement in corneal compression: 77%

SD: standard deviation; UCVA: uncorrected visual acuity; BCLCVA: best contact lens corrected visual acuity; HOA: high ocular aberration; PKP: penetrating keratoplasty. Mild KC (average sim *K* < 45 D). Moderate (average sim *K* 45–52 D). Severe (average sim *K* > 52 D).

**Table 4:** Description of the main studies in keratoconus (KC) management with piggyback contact lens (PBCL) system.

Author	Type of study	Type of SCL	Type of RGPCL	DK (SCL/RGPCL) (barriers)	SLC power (D)	Sample size (eyes)	Severity KC (number of eyes)	BCLCVA (logMAR) (mean)	Follow-up (months) (mean)	Age (mean) (years)	Sex (female/male)
O'Donnell and Maldonado-Codina [94]	Case report	SCL SiH Lotrafilcon A (Focus Night & Day)	High DK RPG (Menicon Z)	140/163	+3.00	Both eyes	Moderate (2)	0.00	3	54	Female
Smith and Carrell [103]	Case report	SCL SiH Balafilcon A (PureVision)	Paflufocon B (Paragon Vision Sciences, Mesa, AZ)	101/58	−12.00	Both eyes	Severe (2)	0.00	6	41	Male
Sengor et al. [92]	Prospective	SCL SiH Lotrafilcon A (Focus Night & Day)	Fluorosilicone Methacrylate Copolymer (Conflex keratoconus 100 UV, Germany)	140/100	+4.00 to −3.50	29	Mild (3) Moderate (16) Severe (10)	0.08	9	28.3	10/6
Acar et al. [97]	Prospective	SCL SiH Balafilcon A (PureVision)	Fluorosilicone Methacrylate Copolymer (CFA 100 UV, Germany)	101/100	NR	14	NR	NR	6	26.3	6/6
Romero-Jimenez et al. [96]	Prospective nondispensing masked	SCL SiH Senofilcon A (Johnson & Johnson, Vision Care Inc., FL, USA)	Tisifilcon A (Rose K2) (Menicon Co. Ltd., Nagoya, Japan)	NR	−6.00 −3.00 +3.00 +6.00	30	Mild (21) Moderate (9)	NR	NR	34.9	5/11

DK: oxygen transmissibility; SCL: soft contact lens; RGPCL: rigid gas permeable contact lens; BCLCVA: best contact lens corrected visual acuity; NR: not reported. Mild KC (average sim *K* < 45 D). Moderate (average sim *K* 45–52 D). Severe (average sim *K* > 52 D).

**Table 5:** Description of the main studies in keratoconus (KC) management with hybrid contact lens (HCL) system.

Author	Type of study	Type of CL	Sample size (eyes)	Severity KC	BCLCVA (logMAR) (mean)	Follow-up (months) (mean)	Mean age or age (years)	Sex (female/male)
Ozkurt et al. [108]	Retrospective case series	SoftPerm CL	24	NR	Better than +0.5	23	25.78	8/6
Nau [107]	Retrospective	SynergEyes HCLs versus RPGCLs	79	NR	+0.2	3	36.17	32/21
Abdalla et al. [112]	Retrospective	SynergEyes HCLs	61	Moderate and severe	0.0 To +0.1 (31.1%) +0.2 to +0.3 (62.3%)	7.8	40	18/26
Carracedo et al. [113]	Prospective comparative	ClearKone HCLs versus patient habitual CL	33	Moderate and severe	−0.024	1	26.3	5/13
Hahemi et al. [106]	Comparative case series	ClearKone HCLs (group 1) versus RPGCLs (group 2)	40	Moderate	Group 1 : 0.01 Group 2 : 0.02	2	Group 1: 33.71 Group 2: 32.16	21/19

BCLCVA: best contact lens corrected visual acuity; NR: not reported; OD: right eye; CL: contact lens. Mild KC (average sim *K* < 45 D). Moderate (average sim *K* 45–52 D). Severe (average sim *K* > 52 D).

**Table 6:** Description of the main studies in keratoconus (KC) management with scleral contact lenses (SsCLs).

Author	Type of study	Sample size (eyes)	Follow-up	Age (years) (mean)	Sex (female/male)	Diameter of lens (mm)	UCVA (logMAR)	BCLCVA (logMAR) (mean)
Carracedo et al. [131]	Prospective comparative	26	8 hours	36.95	12/14	16.5	NR	0.17
Visser et al. [123]	Prospective case series	213	3 weeks–1 year	47.7	64/80	18.5–19.5 (19%) 20.0 (76%) 20–5-21.5(5%)	NR	≤0.1 in 62.9%
Baran et al. [124]	Prospective case series	118	6 months	44	26/33	NR	0.55	≤0.3 in 93%
Stason et al. [127]	Prospective case series	141	6 months	44	NR	NR	≤0.2 in 30% 0.3–0.5 in 32% ≥0.55 in 38%	Improved by 2 or more lines in 55%
Visser et al. [128]	Prospective case series	284	5 months	45	NR	18–25	0.7	0.15

UCVA: uncorrected visual acuity; BCLCVA: best contact lens corrected visual acuity; NR: not reported.

**Table 7:** Contact-lens related complications (adverse events) in keratoconus eye.

Complications	Type of contact lens used	Rate (% eyes)
Discomfort/lens intolerance	RGPCL	3.9 to 32.7 [13, 35, 41]
	HCL	62.5 [112], 12.1 [113], 1.6 [107], 29.1 [108]
Unsatisfactory vision	RGPCL	15.6 [13]
	HCL	5 [112]
Corneal warpage	RGPCL	77 [33]
Alterations of the tear film	RGPCL	[68]
	ScCL	[131], 45 [125]
Difficulties with the lens manipulation	RGPCL	3.1 [13]
	HCL	12.1 [113]
	ScCL	[121]
Neovascularization	SCL SiH	3.2 [20]
Staining	SCL SiH [20]	4.26 [20]
	RGPCL	38 [139]
Injection	SCL SiH	2.13 [20]
	RGPCL	15 [139]
Superficial punctate keratitis	RGPCL	4.6 [13]
		0.002 [41]
Giant papillary conjunctivitis	RGPCL	11.8 [41]
	PBCL	4.6, 13.7 [92]
	HCL	11.5 [112], 25 [108]
Corneal erosion	RGPCL	7.5 [41]
	HCL	1.6 [112]
Corneal staining/SPK	PBCL	3.4 [92]
	HCL	3.2 [112]
Corneal vascularization	HCL	25 [108]
Lens damage/loss	PBCL	3.4 [92]
	HCL	3.2 [112], 29.1 [108]
Interleukin changes	PBCL	58.3 [97]
Keratocyte density	PBCL	58.3 [97]
Discontinuation CLs	HCL	9.1 [113]
Dry eye	HCL	4.9 [112], 4.7 [107]

SCL: soft contact lens; RGPCL: rigid gas permeable contact lens; PBCL: piggyback contact lens system; HCL: hybrid contact lenses; ScCL: scleral contact lens
